# Congenital Asplenia Interrupts Immune Homeostasis and Leads to Excessive Systemic Inflammation in Zebrafish

**DOI:** 10.3389/fcimb.2021.668859

**Published:** 2021-06-28

**Authors:** Lang Xie, Zheyu Chen, Hui Guo, Yixi Tao, Xiaomin Miao, Ronghua Wu, Yun Li

**Affiliations:** ^1^ Institute of Three Gorges Ecological Fisheries of Chongqing, College of Fisheries, Southwest University, Chongqing, China; ^2^ Key Laboratory of Freshwater Fish Reproduction and Development (Ministry of Education), The Key Laboratory of Aquatic Science of Chongqing, Southwest University, Chongqing, China

**Keywords:** congenital asplenia, transcriptome, excessive inflammation, splenic anti-inflammatory reflex, systemic immunity

## Abstract

Splenectomy or congenital asplenia in humans increases susceptibility to infections. We have previously reported that congenital asplenia in zebrafish reduces resistance to *Aeromonas hydrophila* infection. However, the molecular mechanism of systemic immune response in congenitally asplenic individuals is largely unexplored. In this study, we found that pro-inflammatory cytokines were more highly induced in congenitally asplenic zebrafish than wild-type after pathogenic *A. hydrophila* infection and lipopolysaccharide exposure. In addition, a higher aggregation of apoptotic cells was observed in congenitally asplenic zebrafish than that in wild-type. Next, we examined the transcriptome profiles of whole kidneys from wild-type and congenitally asplenic zebrafish to investigate the effects of congenital asplenia on innate and adaptive immune responses induced by the inactivated *A. hydrophila*. Congenital asplenia inactivated the splenic anti-inflammatory reflex, disrupted immune homeostasis, and induced excessive inflammation as evidenced by the highly induced stress response–related biological processes, inflammatory and apoptosis-associated pathways, and pro-inflammatory cytokines/chemokines in congenitally asplenic zebrafish compared with wild-type after vaccination. In addition, complement component genes (*c3a.1*, *c3a.6*, *c4*, *c6*, and *c9*) and several important immune-related genes (*tabp.1*, *tap1*, *hamp*, *prg4b*, *nfil3*, *defbl1*, *psmb9a*, *tfr1a*, and *sae1*) were downregulated in congenitally asplenic zebrafish. Furthermore, congenital asplenia impaired adaptive immunity as demonstrated by downregulation of biological processes and signaling pathways involved in adaptive immune response after vaccination in congenitally asplenic zebrafish. The expression of *MHCII/IgM* was also significantly reduced in the congenitally asplenic zebrafish when compared with wild-type. Together, our study provides an in-depth understanding of spleen function in controlling immune homeostasis and may offer insight into the pathological response in splenectomized or congenitally asplenic patients after infections.

## Introduction

The spleen is a secondary lymphatic and reticuloendothelial organ that performs several functions related to hemocatheresis, blood cell storage, and immune response against infections as well as extramedullary hematopoiesis (Mebius and Kraal, 2005; Bronte and Pittet, 2013). As an immune-related organ, the spleen is crucial in regulating immune homoeostasis through its ability to link innate and adaptive immunity and its function in protecting against infections. Over the past couple of years, the spleen has been considered an accessory organ because surgically splenectomized patients can live normal lives. However, the impact of splenectomy is equivocal. Studies show that the absence of the spleen, or hyposplenia and splenectomy in humans, often carries an increased risk of overwhelming infections caused by encapsulated bacteria (Thomsen et al., 2009; Ram et al., 2010; Kristinsson et al., 2014). Overwhelming postsplenectomy infection (OPSI) is a serious disease that may rapidly progress to fulminant infection and death in 50% of cases in a short time period for patients suspected to have splenectomy (Dahyot-Fizelier et al., 2013; Sinwar, 2014). Consequently, there has been renewed interest in preserving the spleen in circumstances in which splenectomy is thought to be necessary (Tracy and Rice, 2008).

Asplenia or hyposplenia may occur as a result of splenectomy following disease or as a congenital anomaly. Congenital asplenia is a rare disorder characterized by the absence of a spleen at birth and has been referred to as a primary immune deficiency. Patients with congenital asplenia have increased susceptibility to invasive infections along with a high mortality rate despite aggressive therapy. As with the splenectomized patients, congenitally asplenic individuals are susceptible to overwhelming pneumococcal infection throughout life (Mahlaoui et al., 2011). Studies on congenitally asplenic mice reveal that the generation and survival of B-1a cells, a B cell subset that cooperates with the innate immune system to control early bacterial and viral growth, was impaired, and asplenic mice exhibit slowed IgM production and clearance of injected bacteria, resulting in fatal sepsis (Karrer et al., 1997; Wardemann et al., 2002). Compared with the research on splenectomy, reports on the effects of congenital asplenia are relatively scarce. Thus, the physiological and pathological processes in congenitally asplenic patients under pathogen challenge has become the focus of attention.

Zebrafish (Danio rerio) are used for studying animal and human infectious disease (Novoa and Figueras, 2012) and are an accepted model system for filling an important gap between lower vertebrates and mammals in studies of host–pathogen interactions. The immune system of adult zebrafish is similar to that of mammals, including almost all lymphoid organs and immune cell types (Renshaw and Trede, 2012; van der Vaart et al., 2012). The similarities between zebrafish and mammalian immune systems enable numerous researchers to establish models of bacterial and viral infections in zebrafish. In our previous study, we generated a *tlx1^–/–^* zebrafish homozygous mutant, which exhibits congenital asplenia without any other obvious detected defects. Congenital asplenia reduces resistance to *Aeromonas hydrophila* infection in zebrafish as evidenced by the mortality of the congenitally asplenic zebrafish being significantly increased when compared with wild-type (WT) control after infection (Xie et al., 2019). In addition, with the outbreak of infectious diseases, the research around spleen function in fish immunity has been increasingly valued in recent years. Considering the importance of the spleen in mammals and in fish, the molecular mechanism of systemic immune response in congenitally asplenic individuals under pathogen infection must be fully explored.

In this study, the systemic inflammatory response induced by *A. hydrophila* challenge and lipopolysaccharide (LPS) treatment was investigated in both WT and congenitally asplenic zebrafish. In addition, to gain a full understanding of how congenital asplenia affects the immune response, we employed high-throughput sequencing technology to examine the global transcription profiles of whole kidneys (head-kidney and kidney) from both WT and congenitally asplenic zebrafish injected with an inactivated *A. hydrophila* vaccine at 6 h and 10 d, the time corresponding to the occurrence of innate and adaptive immune responses. Comparative transcriptome analyses were then conducted to identify differentially expressed genes between these groups. Our study provides a better understanding of the effects of congenital asplenia on systemic immunity, and in the meantime, understanding the molecular mechanism of systemic immune response in congenitally asplenic zebrafish after vaccination may contribute to pathological understanding and complication control in splenectomized or congenitally asplenic patients after infections.

## Materials and Methods

### Zebrafish

The WT AB and *tlx1^–/–^* mutant fish (Xie et al., 2019) strains were used in this study. Zebrafish were maintained in flow-through aquaria at 28°C ± 0.5°C with a photoperiod of 14 h light and 10 h dark. Fish were fed twice daily with newly hatched brine shrimp (Brine Shrimp Direct). All animal experiments conformed to the Guide for the Care and Use of Laboratory Animals and were approved by the Institutional Animal Care and Use Committee at Southwest University, China.

### Pathogenic *A. hydrophila* Infection and LPS Exposure

*A. hydrophila* strains, which were used in our previous study, were grown in LB medium for 14 h at 30°C with shaking at 170 rpm. Bacterial cells were harvested *via* centrifugation at 5000 rpm for 5 min and then resuspended in phosphate buffered saline (PBS) (Beyotime, China), and the concentration was first determined *via* a turbidity meter (Qiwei, China). Then, bacteria were serially diluted and counted by plating on LB solid medium. About 10^8^ CFU mL^−1^ bacteria were added to the tank water. Fifty zebrafish larvae, randomly selected at 4 days postfertilization (dpf) from each group (WT or congenitally asplenic zebrafish), were exposed to *A. hydrophila* as an immune challenge. Animals were sampled at 6 h after exposure to *A. hydrophila* for qRT-PCR. Another 100 larvae at 4 dpf from each WT or congenitally asplenic zebrafish were also randomly selected for exposure to LPS (50 μg/mL) for 2 d. To evaluate the differences in inflammatory response among WT and congenitally asplenic zebrafish, animals sampled at 6 h after exposure to LPS were used for qRT-PCR; animals sampled at 2 d after exposure to LPS were used for apoptosis assay. Zebrafish larvae treated with PBS were used as a control group.

### Enzyme-Linked Immunosorbent Assay

Triplicate groups of at least 50 WT and congenitally asplenic larval zebrafish were challenged with *A. hydrophila* and exposed to LPS, respectively. Animals were sampled at 0 (blank control), 12, and 24 h after pathogenic *A. hydrophila* infection and LPS exposure for measurement of immune parameters. Cytokines (e.g., Il1β, Il6, and Tnfα) were detected with enzyme-linked immunosorbent assay (ELISA) kits in accordance with the manufacturer’s protocol. The zebrafish tumor necrosis factor α (Tnf-α), Interleukin 6 (Il-6), and Interleukin 1β (Il-1β) ELISA kits were purchased from Sinobestbio (China). The cytokines were expressed as pg/mg protein.

### Apoptosis Assay

For the apoptosis assay, the *in situ* cell death detection kit (C1089, Beyotime, Shanghai, China) was used in our work. Briefly, samples were collected and fixed with 4% PFA overnight. Next, larvae samples were washed with PBS at least three times and then treated with 1% protease K (without DNase) for 30 min. Subsequently, larvae were rinsed with PBS three times again and then labeled with terminal deoxynucleotidyl transferase reaction mixture for 60 min at 37°C under dark conditions. Finally, samples were washed again with PBS, and then, photographs were taken using a LEICA DM6000 microscope (Leica, Germany).

### Vaccination and Sampling

Because *A. hydrophila* challenge results in high mortality, most of the challenged zebrafish do not survive before the activation of adaptive immunity (data not shown). A previous study shows that the vaccine is able to induce both innate and adaptive immune responses in zebrafish (Zhang et al., 2012). Thus, an inactivated *A. hydrophila* vaccine was used. Briefly, *A. hydrophila* cells were inactivated via addition of 0.3% formalin to the culture, followed by incubation at 4°C for 48 h with gentle agitation. After incubation with formalin, 100 μl of formalin-treated bacteria were plated in LB solid medium and cultured at 30°C for 24 h to ensure the effectiveness of bacterial inactivation. Completely inactivated bacterial cells were washed thrice with PBS. For the vaccination, triplicate groups of 30 WT and congenitally asplenic adult zebrafish were injected with 10 μl of inactivated bacterial cells (10^8^ CFU mL^−1^). After euthanasia with 300 ng/ml MS-222, whole kidneys, liver, intestine, gill, and skin were sampled at 0 (blank control), 6 h, and 10 d following intraperitoneal injection with duplicates of 10 fish in each treatment group. The samples were stored at -80°C until RNA extraction.

### cDNA Library Construction and Illumina Deep Sequencing

Total RNA was extracted using the RNAiso Plus kit (Takara, Japan) according to the manufacturer’s protocol and treated with RNase-free DNase I to remove genomic DNA contamination. For each sample, total RNA content was measured using a SmartSpecTM Plus spectrophotometer (Bio-Rad, Hercules, CA, USA), and the quality was assessed by agarose gel electrophoresis. Thereafter, the quality and quantity of RNA were assessed using a NanoPhotometer^®^ spectrophotometer (IMPLEN, CA, USA) and an Agilent 2100 Bioanalyzer (Agilent Technologies, CA, USA). Each sample must meet the conditions of 28S/18S RNA ratio ≥ 1.8 and RNA integrity number > 8.5 for further processing. The high-quality RNA samples were subsequently submitted to the Sangon Biotech Co. Ltd. (Shanghai, china) for library preparation and sequencing. A total amount of 2 μg RNA per sample was used as input material for the RNA sample preparations. Briefly, mRNA was purified from total RNA using poly-T oligo-attached magnetic beads. First-strand cDNA was synthesized using random hexamer primer and M-MuLV Reverse Transcriptase (RNase H). Second-strand cDNA synthesis was subsequently performed using DNA polymerase I and RNase H. Remaining overhangs were converted into blunt ends *via* exonuclease/polymerase activities. After adenylation of 3’ ends of DNA fragments, the adaptor was ligated to prepare for the library. To select cDNA fragments of preferentially 150~200 bp in length, the library fragments were purified with the AMPure XP system (Beckman Coulter, Beverly, USA). RNA-sequencing data were filtered to remove low-quality reads containing reads with adaptors, reads with >10% of the unknown nucleotides (N), and reads with >50% low-quality (Q-value ≤ 20) bases. Clean reads were mapped to the reference genome (GRCz11) by HISAT2 (version 2.0) with default parameters.

### Expression Analysis

Gene expression values of the transcripts were computed by StringTie (version 1.3.3b). Principal component analysis (PCA) and principal coordinate analysis were performed to reflect the distance and difference between samples. The transcripts per million (TPM) eliminates the influence of gene lengths and sequencing discrepancies to enable direct comparison of gene expression between samples. DESeq2 (version 1.12.4) was used to determine differentially expressed genes (DEGs) between two samples. Genes were considered as significant differentially expressed if *q*-value < 0.001 and |Fold-Change| > 1.5. Gene Ontology (GO) terms and Kyoto Encyclopedia of Genes and Genomes (KEGG) pathways with false discovery rate (*q*-value) < 0.05 were considered as significantly altered.

### Quantitative RT-PCR (qRT-PCR)

All qRT-PCRs were carried out in a CFX96 Real-Time PCR Detection System (Bio-Rad, USA) in 20-μl reactions containing 10 μl of SsoFast™ EvaGreen^®^ SuperMix (Bio-Rad, USA), 1 μl of diluted cDNA or nuclease-free water as a negative control, 8 μl of nuclease-free water, and 0.5 μl of 10 μM stock solutions of each primer. The PCRs were initiated by denaturation at 95°C for 30 s, followed by 40 amplification cycles of 95°C for 5 s and 60°C for 5 s. The melting curve for the PCR products was generated by heating from 65°C to 95°C in 0.5°C increments with a 5-s dwell time, and a plate read was performed at each temperature. The relative expression levels of the target genes were evaluated using the formula *R* = 2^−ΔΔCt^ with *ef1α* as a reference gene to normalize the expression values. Three biological replicates were performed for each sample. *nfkb1*and *nfkb2* primers were used as described by Wang et al. (2018). *MHCII*, *IgM*, *IgZ1*, and *IgZ2* primers were used as described by Liu et al. (2015). The primer sequences used for the PCRs are listed in **Table S1**.

### Statistical Analysis

Data are expressed as the mean ± SD. Student’s *t*-test and one-way ANOVA followed by the Duncan *post-hoc* test were performed to determine statistical significance at *P* <.05.

## Results

### Effects of Congenital Asplenia on Inflammatory Response in Zebrafish Larva

**Figure 1:**
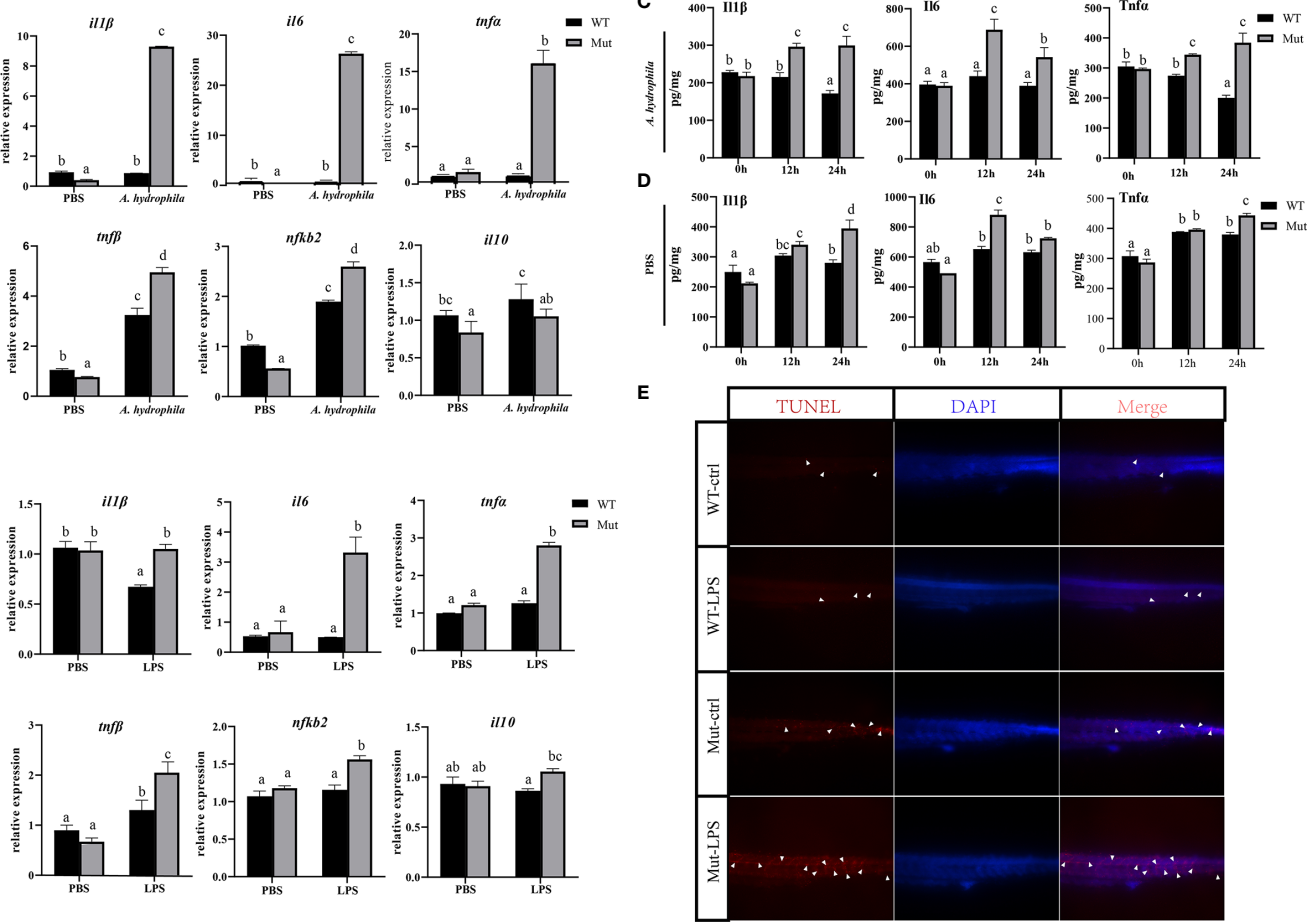
Effects of congenital asplenia on inflammatory response in zebrafish larvae. **(A)** The expression of inflammatory response–related genes after pathogenic *A. hydrophila* infection in zebrafish larvae. **(B)** The expression of inflammatory response–related genes after LPS exposure. **(C, D)** The expression of Il1β, Il6, and Tnfα proteins after *A. hydrophila* challenge and LPS by ELISA. **(E)** A high aggregation of apoptotic cells was observed in the trunk of congenitally asplenic zebrafish by TUNEL assay. The white arrowhead indicates TUNEL-positive cells. Different letters (a, b, c, etc.) indicate significant differences (*p* < .05).

In this part, zebrafish larvae at 4 dpf (mouthopening stage) were exposed to *A. hydrophila.* To evaluate the early immune response in WT and congenitally asplenic zebrafish after pathogen infection, expression levels of several innate immunity-related genes were analyzed at 6 h after *A. hydrophila* challenge. As shown in **Figure 1A**, pro-inflammatory cytokines (*il1β*, *il6*, *tnfα*, and *tnfβ*) and NF-κB subunits (*nfkb2*) were more highly induced in congenitally asplenic zebrafish when compared with WT. However, the expression of the anti-inflammatory cytokine gene (*il10*) failed to upregulate, indicating the uncontrolled inflammatory response in congenitally asplenic zebrafish. To test this hypothesis, inflammatory response was examined in zebrafish larvae after exposure to LPS (activator of the inflammatory response). As shown in **Figure 1B**, except for *il1β*, the expression of *il6*, *tnfα*, *tnfβ*, and *nfkb2* were also found to be more highly induced after LPS treatment as the same expression patterns as in the *A. hydrophila* challenge. Then, ELISA was performed to examine the Il1β, Il6, and Tnfα proteins after *A. hydrophila* challenge and LPS exposure. As shown in **Figure 1C**, Il1β, Il6, and Tnfα protein levels were significantly upregulated, and they exhibited higher expression patterns in congenitally asplenic zebrafish when compared with WT after *A. hydrophila* challenge at 12 and 24 h. In addition, Il6 also exhibited significant upregulation and higher expression patterns in congenitally asplenic zebrafish after being exposed to LPS at 12 h. Then, Il1β and Tnfα were more highly induced in congenitally asplenic zebrafish at 24 h post-LPS exposure (**Figure 1D**). Because excessive inflammatory cytokine release possibly induces apoptosis, we further measured the apoptosis after LPS treatment by the TUNEL assay. As shown in **Figure 1E**, an increased apoptosis signal was founded in congenitally asplenic zebrafish when compared with WT before LPS treatment. After exposure to LPS for 2 d, a high aggregation of apoptotic cells was observed in the trunk of congenitally asplenic zebrafish. In contrast, only a few TUNEL-positive cells were found in WT zebrafish after LPS exposure.

### RNA Sequencing, Read Mapping, and Differential Expression Analysis

The adaptive immunity of zebrafish occurs until 4 weeks after fertilization; thereby, adult zebrafish were used in this part. To gain full understanding of the effects of congenital asplenia on systemic immunity, RNA-Seq was applied to compare the innate and adaptive immune response between WT and congenitally asplenic zebrafish in the whole kidney (kidney and head-kidney) tissue. Samples (WT_0h and Mut_0h) were collected before vaccination, and samples (WT_6h and Mut_6h; WT_10d and Mut_10d) were obtained after vaccination for 6 h and 10 d, respectively. The library construction, quality control analyses, and reads mapped information of the 18 libraries are summarized in **Table 1**. In total, these results indicate that the RNA-seq data are of high quality and can be used for further analysis. The PCA score plot shows that the three RNA-seq samples as biological replicates were clustered (**Figure S1**). All the sequencing data were submitted to the Sequence Read Archive in the National Center for Biotechnology Information under the accession numbers PRJNA688352 and PRJNA688655.

**Table 1:** Summary of the sequencing data. Each sample was triplicated as indicated by the number after the hyphen, e.g., WT_0h_k1, WT_0h_k2, WT_0h_k3.

Samples	Read length (bp)	Raw reads	Clean reads	Clean reads ratio (%)	Mapped reads ratio (%)
WT_0h_k1	150	39688256	38648392	97.38%	91.06%
WT_0h_k2	150	47770252	46359928	97.05%	90.61%
WT_0h_k3	150	44502320	43160098	96.98%	90.05%
WT_6h_k1	150	37738388	36603362	96.99%	90.44%
WT_6h_k2	150	51173184	49579150	96.89%	90.87%
WT_6h_k3	150	47965710	46524076	96.99%	90.57%
WT_10d_k1	150	58083772	56355982	97.03%	90.57%
WT_10d_k2	150	42363902	41143198	97.12%	90.75%
WT_10d_k3	150	47730604	46416376	97.25%	90.81%
Mut_0h_k1	150	46365364	44922362	96.89%	91.75%
Mut_0h_k2	150	36954740	36074568	97.62%	91.03%
Mut_0h_k3	150	39276634	38156430	97.15%	91.16%
Mut_6h_k1	150	45707310	44402338	97.14%	90.32%
Mut_6h_k2	150	44093386	42793992	97.05%	90.95%
Mut_6h_k3	150	45474450	44193726	97.18%	91.10%
Mut_10d_k1	150	46041914	44638712	96.95%	91.87%
Mut_10d_k2	150	41090350	39821806	96.91%	92.03%
Mut_10d_k3	150	52393646	50828956	97.01%	91.81%

Based on the following criteria: |Fold-Change| > 1.5 and adjusted *q*-value < 0.001, the number of DEGs was analyzed, and the data are shown in **Figure S2**. The number of DEGs identified in WT-6h_vs_WT-0h was 4388 with 2764 being upregulated DEGs and 1634 being downregulated DEGs. The number of DEGs identified in Mut-6h_vs_Mut-0h was 4494 with 2343 upregulated DEGs and 2153 downregulated DEGs. The number of DEGs identified in WT-10d_vs_WT-0h was 1681 with 1075 being upregulated DEGs and 606 being downregulated DEGs, and the number of DEGs in Mut-10d_vs_Mut-0h was 1231 with 197 upregulated DEGs and 1034 downregulated DEGs. When comparing the Mut group with the WT group, the number of DEGs in Mut-6h_vs_WT-6h was 1735 with 846 DEGs being upregulated and 889 DEGs being downregulated in the Mut group; the number of DEGs in Mut-10d_vs_WT-10d was 1963 with 640 DEGs being upregulated and 1323 DEGs being downregulated in the Mut group. Then, all of the DEGs with significant change were subject to further GO and KEGG enrichment analysis.

### Zebrafish With Congenital Asplenia Are More Hyperresponsive After Vaccination

**Figure 2:**
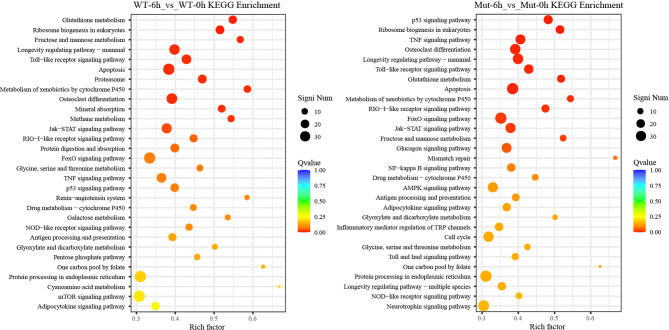
The top 30 enriched KEGG pathways in WT-6h_vs_WT-0h and Mut-6h_vs_Mut-0h. The color and size of the dots indicate Q-values and DEG numbers in pathways, respectively.

To investigate whether vaccination could successfully induce immune response in WT and asplenic fish, comparative transcriptome analysis was performed on WT-6h_vs_WT-0h and Mut-6h_vs_Mut-0h. In KEGG analysis, the top 30 enriched pathways of WT-6h_vs_WT-0h and Mut-6h_vs_Mut-0h are shown in **Figure 2**. The KEGG enrichment analysis showed that 14 and 12 pathways were significantly affected in asplenic and WT zebrafish, respectively. Both of the two contrasts clustered several conserved immune-related pathways, including the Jak-STAT and toll-like receptor signaling pathways and glutathione metabolism. The P53 signaling pathway was uniquely enriched as the topmost represented KEGG categories in Mut-6h_vs_Mut-0h. In addition, the TNF, RIG-I-like receptor, and FoxO signaling pathways were also uniquely and significantly enriched in Mut-6h_vs_Mut-0h (**Figure 2**). Detailed information of significantly enriched KEGG pathways in WT-6h_vs_WT-0h and Mut-6h_vs_Mut-0h is listed in **Table S2**.

**Figure 3:**
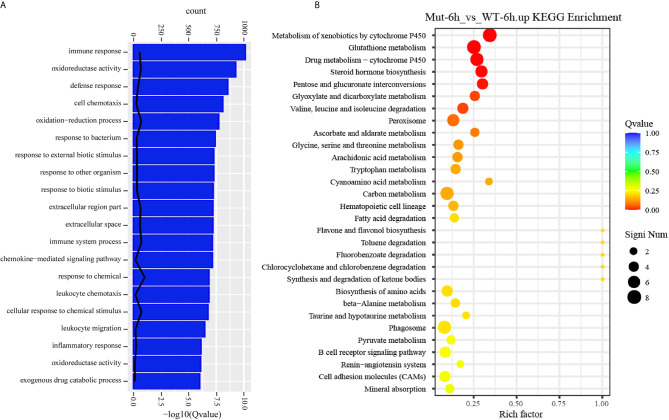
GO functional enrichment and KEGG analysis from upregulated DEGs in Mut-6h_vs_WT-6h. **(A)** The top 20 significantly upregulated GO categories in Mut-6h_vs_WT-6h. The *X*- and *Y*-axes represent the significantly enriched GO terms and the corresponding number of DEGs, respectively. **(B)** The top 30 upregulated KEGG pathways in Mut-6h_vs_WT-6h. The numbers of DEGs in each pathway are counted and rich factors and *P*-values are displayed.

To gain a comprehensive understanding of the mechanisms involved and to directly understand the effects of congenital asplenia on innate immunity in zebrafish, comparative transcriptome analyses were further performed on the DEGs of Mut-6h_vs_WT-6h. For the GO enrichment analysis, most of the GO terms that were significantly enriched from the upregulated DEGs were involved in early stress response, including defense response, cell chemotaxis, response to bacterium, response to external biotic stimulus, response to other organism, response to biotic stimulus, and chemokine-mediated signaling pathway as well as response to chemical, leukocyte chemotaxis, leukocyte migration, and inflammatory response (**Figure 3A**). The complete information of enriched GO terms from the upregulated DEG in Mut-6h _vs_WT-6h is listed in **Table S3**. In KEGG analysis, KEGG categories significantly enriched from the upregulated genes in Mut-6h_vs_WT-6h describe metabolism of xenobiotics by cytochrome P450, glutathione metabolism, drug metabolism–cytochrome P450, steroid hormone biosynthesis, pentose and glucuronate interconversions, and valine, leucine and isoleucine degradation (**Figure 3B** and **Table S4**).

### Congenital Asplenia Induces Excessive Systemic Inflammation After Vaccination in Zebrafish

**Figure 4:**
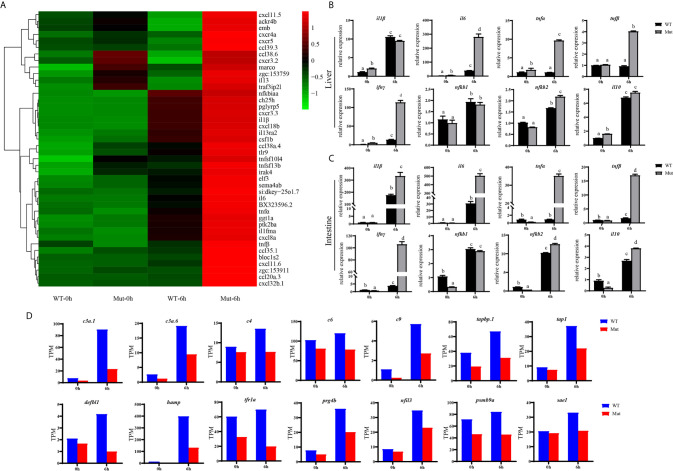
Congenital asplenia induces excessive systemic inflammation after vaccination in zebrafish. **(A)** The expression level of inflammatory cytokines/chemokines displayed with a heat map. **(B)** The expression of inflammation-related genes in liver after vaccination by qRT-PCR. **(C)** The expression of inflammation-related genes in intestine after vaccination by qRT-PCR. **(D)** The expression level of other important immune-related genes by RNA-seq. Different letters (a, b, c, etc.) indicate significant differences (*p* < .05).

The TNF and RIG-I-like receptor signaling pathways were major signaling pathways involved in inflammatory response, and both of them were uniquely and significantly enriched in Mut-6h_vs_Mut-0h (**Figures S3**, **S4**).We hypothesized that vaccination might induce excessive inflammation in asplenia zebrafish. To understand the differences of inflammatory response in WT and congenitally asplenic zebrafish, inflammatory cytokines/chemokines were identified. Our RNA-seq analysis revealed that most regulated pro-inflammatory inflammatory cytokines/chemokines (*il1β*, *il6*, *tnfα*, *tnfβ*, *il13*, *il13ra2*, *cxcr4a*, *elf3*, *cxcl8a*, *cxcl18b*, *csf1b*, *ccl20a.3*, *ccl39.3*, etc.) are more highly induced in congenitally asplenic zebrafish than WT following vaccination (**Figure 4A**). The expression patterns of regulated cytokines/chemokines from all other groups are displayed in **Figure S5**. To confirm the excessive inflammatory response in congenitally asplenic zebrafish, the expression pattern of pro-inflammatory cytokines (*il1β*, *il6*, *tnfα*, *tnfβ*, and *ifnγ*), NF-κB subunits (*nfkb1* and *nfkb2*), and an anti-inflammatory cytokine (*il10*) was also identified in liver and intestine tissue. In liver, *il6*, *tnfα*, *tnfβ*, *ifnγ*, and *nfkb2* expression were more highly induced in congenitally asplenic zebrafish (**Figure 4B**). In intestine, the expression level of *il1β*, *il6*, *tnfα*, *tnfβ*, *ifnγ*, and *nfkb2* were also significantly higher in congenitally asplenic zebrafish when compared with WT (**Figure 4C**).

Other important immune-related genes were also identified. As shown in **Figure 4D**, genes encoding complement components, including *c3a.1*, *c3a.6*, *c4*, *c6*, and *c9* are downregulated in asplenic fish when compared with WT. In addition, other genes, including *tabp.1*, *tap1*, *hamp*, *prg4b*, *nfil3*, *defbl1*, *psmb9a*, *tfr1a*, and *sae1*, which are crucial for immune response, are also downregulated in asplenia zebrafish compared with WT.

### Congenital Asplenia Results in an Impaired Adaptive Immune Response After Vaccination in Zebrafish

**Figure 5:**
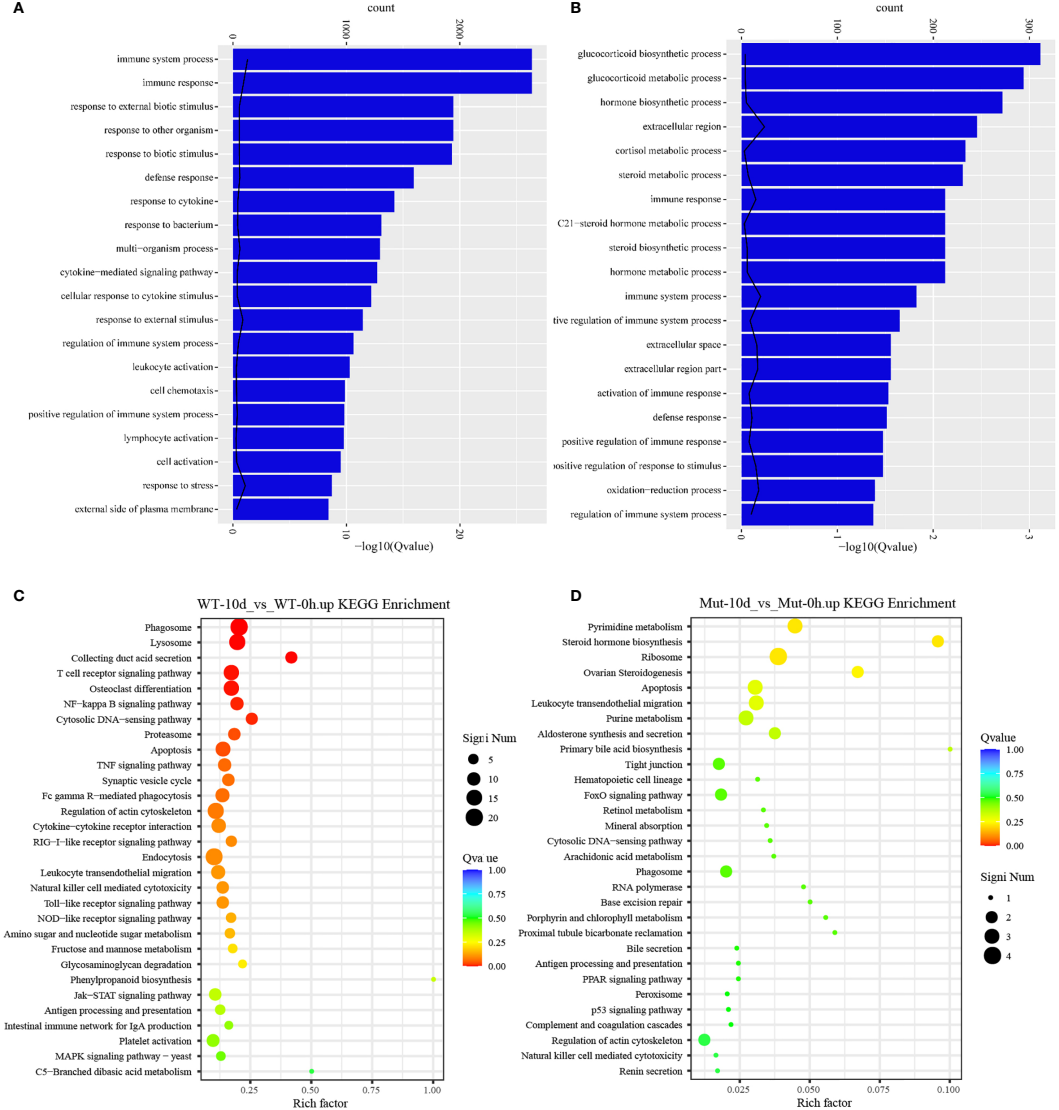
GO functional enrichment and KEGG analysis from upregulated DEGs in WT-10d_vs_WT-0h and Mut-10d_vs_Mut-0h. **(A, B)** The top 20 significantly upregulated GO categories in WT-10d_vs_WT-0h and Mut-10d_vs_Mut-0h, respectively. The *X*- and *Y*-axes represent the significantly enriched GO terms and the corresponding number of DEGs, respectively. **(C, D)** The top 30 upregulated KEGG pathways in WT-10d_vs_WT-0h and Mut-10d_vs_Mut-0h, respectively. The numbers of DEGs in each pathway are counted, and rich factors and *P*-values are displayed.

**Figure 6:**
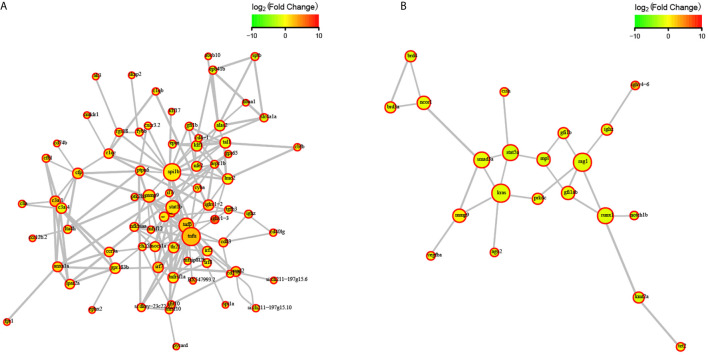
STRING network analysis on DEGs annotated in GO: immune system process. **(A)** WT-10d_vs_WT-0h. **(B)** Mut-10d_vs_Mut-0h. The nod represents proteins, bigger means more interaction with others. The red color means upregulated DEGs, and the green means downregulated DEGs.

To explore the effects of congenital asplenia on adaptive immunity in zebrafish, comparative transcriptome analysis was performed on the DEGs of WT-10d_vs_WT-0h and Mut-10d_vs_Mut-0h. For the GO enrichment analysis, a total of 350 GO terms significantly enriched from the upregulated genes were identified, and the top 20 upregulated GO terms in WT-10d_vs_WT-0h are shown in **Figure 5A**. The GO terms of the immune system process, immune response, response to external biotic stimulus, response to other organisms, response to biotic stimulus, defense response, and response to cytokines were significantly enriched (**Figure 5A**). In addition, 31 adaptive immune response–related GO terms (T cell activation, B cell mediated immunity, B cell activation, lymphocyte-mediated immunity immunoglobulin production, etc.) were also significantly upregulated in WT-10d_vs_WT-0h (**Table S5**). The complete GO terms significantly enriched from the upregulated genes in WT-10d_vs_WT-0h are shown in **Table S6**. However, only 28 GO terms were significantly enriched from the upregulated genes in Mut-10d_vs_Mut-0h, and most of them were more often associated with hormone/steroid biosynthetic and metabolic process as well as several immune-related biological process (**Figure 5B**). The complete information of the significantly upregulated GO terms in Mut-10d_vs_Mut-0h are shown in **Table S7**. In KEGG analysis, pathways significantly enriched from the upregulated genes in WT-10d_vs_WT-0h include phagosome, lysosome, collecting duct acid secretion, osteoclast differentiation, T cell receptor signaling pathway, NF-kappa B signaling pathway, cytosolic DNA-sensing pathway, apoptosis, and proteasome (**Figure 5C** and **Table S8**). However, no immune-related pathways significantly enriched from upregulated genes were identified in Mut-10d_vs_Mut-0h (**Figure 5D**). The protein–protein interactions based on DEGs annotated in the immune system process of two vaccination groups also displayed a different immune response interaction network (**Figure 6**).

**Figure 7:**
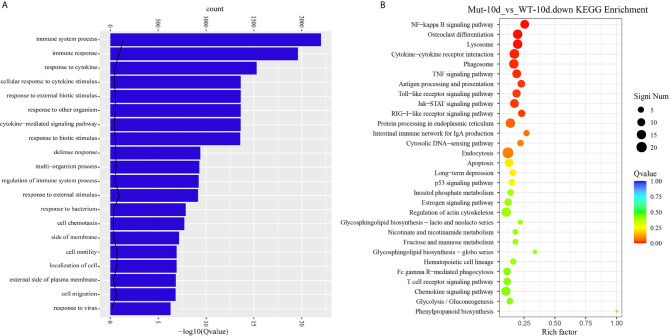
GO functional enrichment and KEGG analysis from downregulated DEGs in Mut-10d_vs_WT-10d. **(A)** The top 20 significantly downregulated GO categories in Mut-10d_vs_WT-10d. The *X*- and *Y*-axes represent the significantly enriched GO terms and the corresponding number of DEGs, respectively. **(B)** The top 30 downregulated KEGG pathways in Mut-10d_vs_WT-10d. The numbers of DEGs in each pathway are counted, and rich factors and *P*-values are displayed.

Next, comparative transcriptome analysis of DEGs of Mut-10d_vs_WT-10d were done to further investigate the difference of adaptive immune responses in WT and asplenic zebrafish after vaccination. GO functional enrichment analysis indicated that GO terms, such as immune system process, immune response, response to cytokine, response to bacterium, and defense response, were significantly downregulated in asplenia zebrafish (**Figure 7A**). In addition, multiple adaptive immunity–associated GO terms (lymphocyte activation, T cell activation, regulation of T cell activation, negative regulation of T cell activation, positive regulation of T cell proliferation, positive regulation of lymphocyte proliferation, positive regulation of lymphocyte activation, regulation of T cell proliferation, regulation of lymphocyte proliferation, lymphocyte costimulation, positive regulation of T cell activation, T cell costimulation, T cell proliferation, lymphocyte proliferation, etc.) were significantly downregulated in congenital asplenic zebrafish (**Table 2**). The complete information of significantly downregulated GO terms in Mut-10d_vs_ WT-10d are shown in **Table S9**. In KEGG analysis, NF-kappa B signaling pathway, lysosome, osteoclast differentiation, cytokine-cytokine receptor interaction, TNF signaling pathway, phagosome, antigen processing and presentation, Jak-STAT signaling pathway, Toll-like receptor signaling pathway, and RIG-I-like receptor signaling pathway were significantly downregulated in congenital asplenic zebrafish (**Figure 7B** and **Table S10**).

**Table 2:** The significantly downregulated GO terms involved in adaptive immunity in Mut-10d_vs_WT-10d.

GO.ID	Term	Ontology	Significant	Annotated	*P*-value	*Q*-value
GO:0046649	lymphocyte activation	biological process	22/917	133/20731	8.80E-08	2.67E-05
GO:0042110	T cell activation	biological process	15/917	66/20731	1.40E-07	3.63E-05
GO:0050863	regulation of T cell activation	biological process	8/917	33/20731	7.40E-05	0.006453
GO:0050863	regulation of T cell activation	biological process	8/917	33/20731	7.40E-05	0.006453
GO:0050868	negative regulation of T cell activation	biological process	6/917	20/20731	0.00017	0.01143
GO:0042102	positive regulation of T cell proliferation	biological process	5/917	15/20731	0.00035	0.020149
GO:0050671	positive regulation of lymphocyte proliferation	biological process	5/917	15/20731	0.00035	0.020149
GO:0051251	positive regulation of lymphocyte activation	biological process	10/917	62/20731	0.00036	0.02059
GO:0042129	regulation of T cell proliferation	biological process	6/917	23/20731	0.00039	0.02147
GO:0050670	regulation of lymphocyte proliferation	biological process	6/917	23/20731	0.00039	0.02147
GO:0031294	lymphocyte costimulation	biological process	4/917	9/20731	0.0004	0.021483
GO:0050870	positive regulation of T cell activation	biological process	5/917	16/20731	0.00049	0.024804
GO:0031295	T cell costimulation	biological process	4/917	9/20731	0.0004	0.021482
GO:0042098	T cell proliferation	biological process	6/917	24/20731	0.0005	0.025023
GO:0048534	hematopoietic or lymphoid organ development	biological process	35/917	438/20731	0.00057	0.027892
GO:0046651	lymphocyte proliferation	biological process	6/917	27/20731	0.00098	0.040909

### Experimental Validation of DEGs

**Figure 8:**
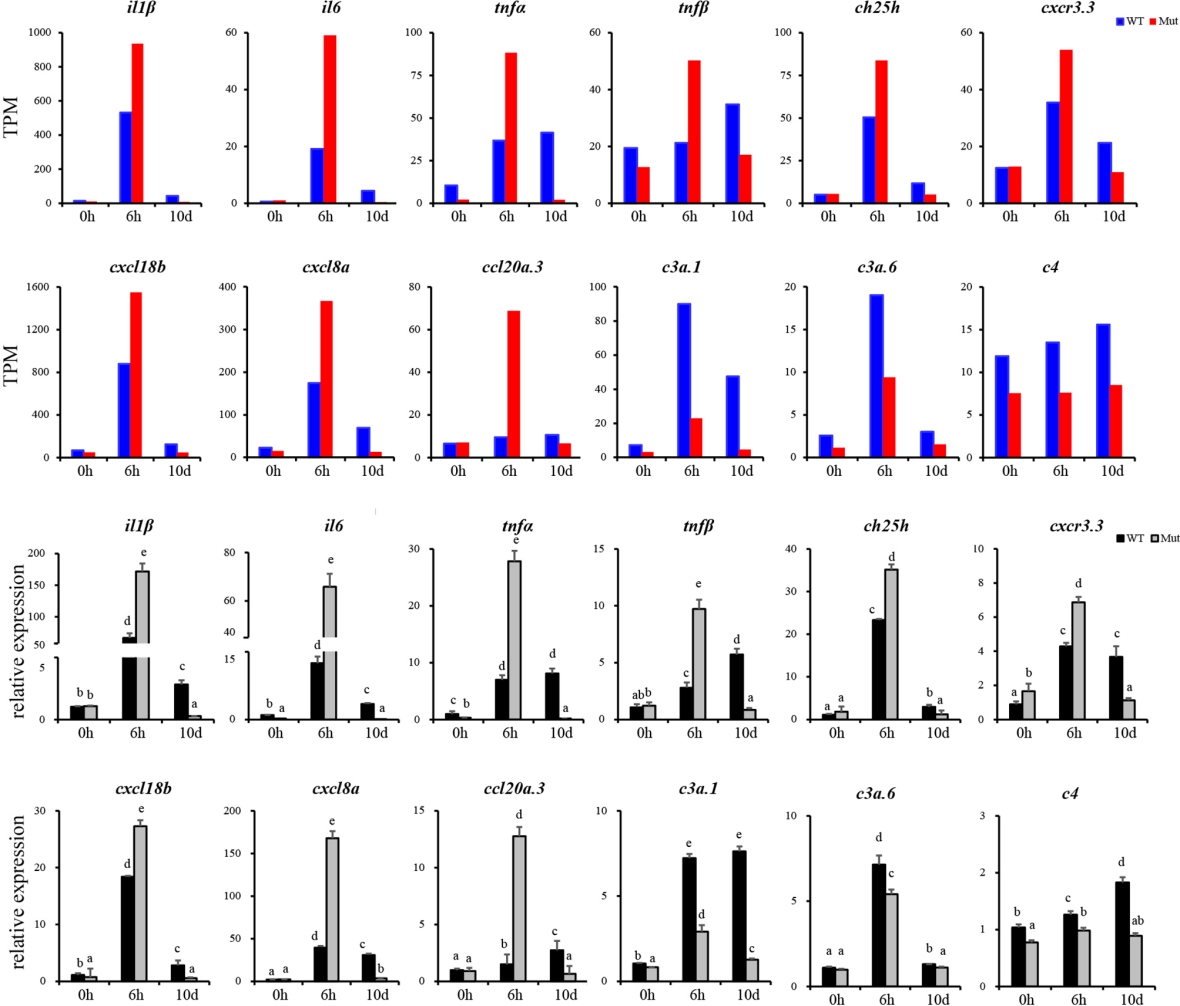
Validation of the 12 DEGs by qRT-PCR. WT animals and congenitally asplenic zebrafish sampled at 0 h were used as controls. The data were normalized to the expression level of WT-0h. The data are reported as the mean ± SEM. Different letters (a, b , c etc.) indicate significant differences (*p* <.05).

**Figure 9:**
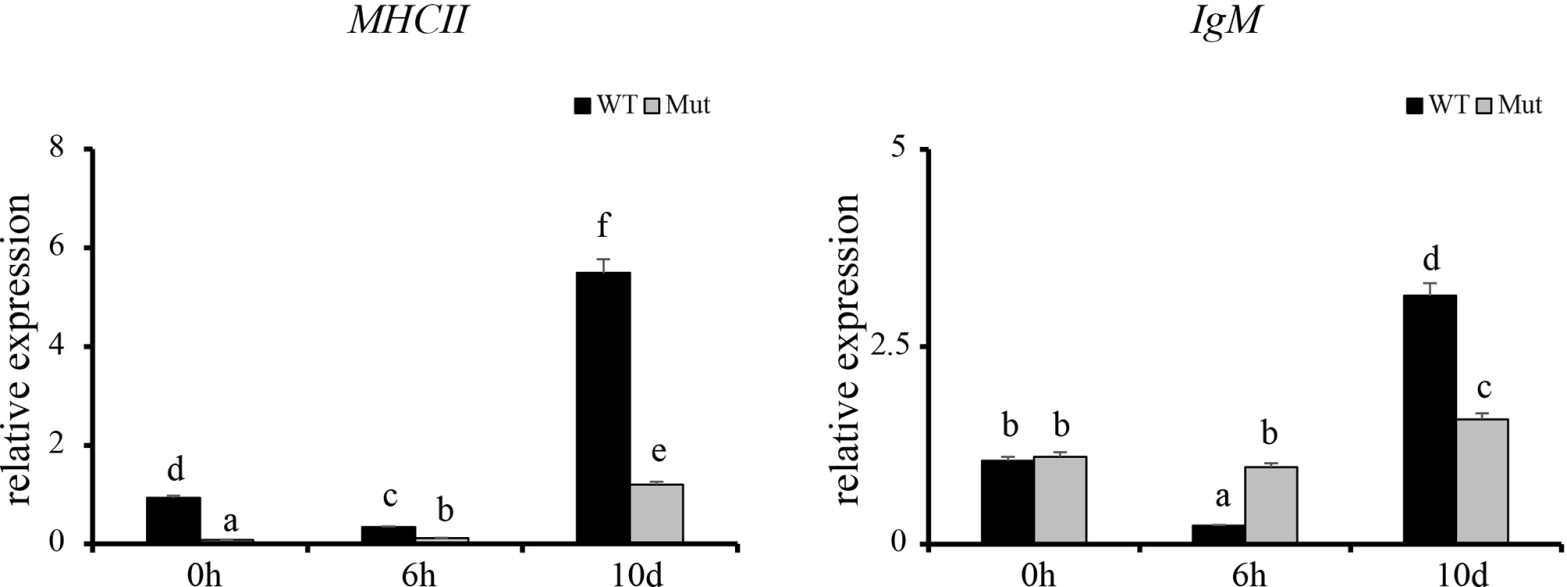
Expression profiles of *MHCII and IgM* after vaccination in whole kidneys. WT animals and congenitally asplenic zebrafish sampled at 0 h were used as controls. The data were normalized to the expression level of WT-0h. The data are reported as the mean ± SEM. Different letters (a, b, c, etc.) indicate significant differences (*p* < .05).

To validate the results of RNA-seq, 12 immune response–related genes, including *il1β*, *il6*, *tnfα*, *tnfβ*, *ch25h*, *cxcr3.3*, *cxcl8a, cxcl18b, ccl20a.3*, *c3a.1*, *c3a.6*, and *c4* were chosen for qPCR detection. The gene expression patterns detected by qPCR were similar to those obtained from the RNA-seq results (**Figure 8**). Furthermore, expression of *MHCII* and *IgM* were also identified to compare the changes in adaptive immune responses in WT and asplenic zebrafish. The expression of *MHCII* exhibited a higher expression level in WT animals compared with its level in the asplenic zebrafish. *IgM* showed upregulation, and its expression reached the highest level in the 10 days postvaccination in both WT animals and asplenic zebrafish, and its expression was significantly reduced in the asplenic zebrafish compared with its expression in WT animals (**Figure 9**).

## Discussion

Although the spleen is the largest immune organ in the human body, it has long been ignored until more and more surgically splenectomized patients and congenitally asplenic individuals were found to have immune-related deficiencies. In recent years, a growing number of studies have been conducted on the role of the spleen in inflammation. However, its role in controlling the systemic inflammatory response is still controversial. It is reported that the spleen is the main source of TNF production, and rats with splenectomy reduced the levels of inflammatory cytokines and leukocyte infiltration and developed acute pancreatitis more slowly than rats without splenectomy (Zhou et al., 2019). On the contrary, splenectomized mice with acute kidney injury resulted in increased serum inflammatory cytokines and worse lung injury as evidenced by the higher lung chemokines and the increased lung capillary leak (Andres-Hernando et al., 2011), implying the spleen may also play roles in downregulating the inflammatory response. Hence, it is necessary to establish a new model to explain the role of the spleen in inflammation. In our previous study, we generated a congenitally asplenic zebrafish model that was more susceptible to *A. hydrophila* infection (Xie et al., 2019). In this study, the inflammatory response of the congenitally asplenic zebrafish was identified after pathogenic *A. hydrophila* infection and LPS exposure. We found that pro-inflammatory cytokines and NF-κB subunits were more highly induced in congenitally asplenic zebrafish after exposure to *A. hydrophila* and LPS. Consistent with the mRNA expression, Il1β, Il6, and Tnfα proteins also exhibited higher expression patterns in congenitally asplenic zebrafish after exposure to *A. hydrophila* and LPS. It is reported that cytokines and chemokines, including *il1β*, *il6*, and *tnfα* are main pro-inflammatory factors involved in regulation of inflammation, and the excessive production of these molecules can be deleterious and associated with disease progression, inflammatory disorders, tissue damage, and apoptosis (Tracey, 2002; Takeshita and Ransohoff, 2012; Bleau et al., 2016; Hasegawa et al., 2017; Kany et al., 2019). Thus, our study suggested that congenital asplenia might induce uncontrolled inflammatory response. In addition, an apoptosis assay revealed the inflammatory injury in congenitally asplenic zebrafish. Taken together, our results suggested the anti-inflammatory effects of spleen in zebrafish as with the study in mice (Andres-Hernando et al., 2011).

In teleosts, the head-kidney and spleen are the major sites producing immune complexes and responsible for the trapping and eliminating pathogens from the whole circulation (Espenes et al., 1996; Hansen and Zapata, 1998). To gain full understanding of how congenital asplenia affects the systemic immune response, we performed comparative transcriptome analysis of whole kidneys (head-kidney and kidney) from both WT and congenitally asplenic zebrafish at the time points corresponding to the occurrence of innate and adaptive immunity after vaccination. According to KEGG enrichment analysis, several conserved immune-related pathways were upregulated in both WT and asplenic zebrafish by 6 h postvaccination, indicating the vaccination successfully activated the immune system. Interestingly, we found that zebrafish with congenital asplenia were more hyperresponsive as evidenced by the highly reduced stress response–associated biological processes (e.g., cell chemotaxis, leukocyte chemotaxis, leukocyte migration, inflammatory response) in asplenic zebrafish after vaccination when compared with WT. Thus, our data indicate that congenital asplenia unbalances the transcriptional regulation in early stress response.

Inflammation is an immune system response to external physical or chemical stimuli or bacterial infection, and it is usually considered a defensive reaction and protects the host from pathogens. However, excessive or uncontrolled systemic inflammation can disrupt immune function and aggravate disease status (Cohen, 2002; Calder et al., 2009). In RNA-seq analysis and qRT-PCR in liver and intestine, pro-inflammatory cytokines/chemokines were more highly induced in asplenic zebrafish. In accordance with the overproduction of these cytokines/chemokines, the TNF and RIG-I-like receptor signaling pathways were uniquely and significantly activated in asplenic zebrafish. It is well documented that TNF signaling pathway is one of the major signaling pathways involved in inflammatory response, and optimal regulation of TNF signaling is essential to maintain tissue homeostasis and prevent inflammatory pathology (Brenner et al., 2015; Van Quickelberghe et al., 2018). The RIG-I-like receptor signaling pathway is known to be essential for triggering inflammatory response upon infections (Zhao et al., 2020). Thus, our data indicate that congenital asplenia induces excessive inflammation after vaccination in zebrafish. In addition, we only observed the activation of the p53 signaling pathway in asplenic zebrafish (**Figure S6**), which is one of the major pathways to induce apoptosis (Wang et al., 2015). Combined with the characteristic of hyperresponsiveness to stress, overproduction of cytokines/chemokines and activation of inflammatory-related signaling pathways, our data suggest congenital asplenia might disrupt immune homeostasis and induce inflammatory damage in zebrafish, which is consistent with the experiment described above.

Splanchnic anti-inflammatory reflex has been described as a powerful mechanism involved in the suppression of inflammatory response in mammals. During systemic inflammation, the production of pro-inflammatory mediators is down-modulated by this reflex, which requires the activation of neural circuits (Tracey, 2002; Martelli et al., 2014b). It is well accepted that the splanchnic anti-inflammatory reflex relies on efferent neural connections from the brain to the viscera, especially the spleen (known as the splenic anti-inflammatory reflex), reducing the production of pro-inflammatory cytokines (Okusa et al., 2017). In rats, splenectomy disrupts the efferent neural connection to the brain and inactivates the splenic anti-inflammatory pathway, leading overproduction of circulating cytokines during systemic inflammation (Huston et al., 2006; Martelli et al., 2014a). Our data suggest that the overproduction of cytokines/chemokines and excessive inflammatory response in congenitally asplenic zebrafish might result from inactivation of splenic anti-inflammatory reflex caused by congenital asplenia. In addition, our study is the first to present findings that the splenic anti-inflammatory reflex plays an essential role in the regulation of inflammation in fish.

Other important immune-related genes were also identified to study the effects of congenital asplenia on innate immunity. It is reported that the complement system plays essential roles in microbial killing, phagocytosis, antibody production, and cell lysis (Boshra et al., 2006). Children with asplenia/hyposplenism have severe combined immune deficiency, including complement C2 deficiency (Scheuerman et al., 2014). In the present study, a number of complement components including *c3a.1*, *c3a.6*, *c4*, *c6*, and *c9* showed a reduced expression level in the congenitally asplenic zebrafish, indicating that the complement system was partially impaired. *Defbl1* (also known as *β-def-1*) is a crucial antimicrobial peptide that plays an essential role in protecting the intestine against infection as a result of antibacterial and immunomodulatory properties (Cederlund et al., 2011). *Hamp* is able to inhibit growth of certain pathogenic microorganisms, including *E. coli*, *V. anguillarum*, *S. aureus*, and *B. subtilis* (Lin et al., 2014). In our present study, *defbl1* and *hamp* were reduced in congenitally asplenic zebrafish compared with WT. In addition, *tap1* and *tapbp.1*, which are involved in antigen processing and presentation (Xu et al., 2017), were also reduced in congenitally asplenic zebrafish. Thus, our data suggest congenital asplenia might impair the antibacterial ability in zebrafish. Hematopoiesis is the synthesis and development of all types of immune cells. In this study, hematopoiesis-related genes, including *tfr1a*, *gfi1b*, *nfil3*, *prg4b*, and *sae1* were downregulated in asplenia zebrafish compared with WT. All of these results indicate that congenital asplenia might impair the fish immune system.

Establishment of adaptive immunity needs 1–2 weeks and is important for host defense during the latter phases of an infection (Farber et al., 2016). The adaptive immune response is associated with the activation of lymphocytes and adaptive immune mechanisms, which allows specific recognition and elimination of the pathogen (Farber et al., 2016). Previous studies reveal that mice with congenital asplenia display impaired B-1a cell generation and delayed or lower IgM antibody secretion (Karrer et al., 1997; Wardemann et al., 2002). According to GO and KEGG enrichment analysis, adaptive immune response–associated biological processes and signaling pathways were highly activated in WT after vaccination although they showed reduced activation or failed to activate in congenitally asplenic zebrafish, indicating that the adaptive immunity was impaired by congenital asplenia. In addition, zebrafish with congenital asplenia displayed a low expression level of *MHCII/IgM*. In fish, antigen-presenting cells ingest, process, and present antigens for T cells *via MHCII*. IgM is the first antibody produced during the immune response, and it provides a crucial line of defense in the immune system. Therefore, our data suggest that antigen processing and presentation/antibody secretion might be retarded due to the congenital asplenia, which is consistent with previous research in mice (Karrer et al., 1997). Splenectomized or congenitally asplenic patients often have an increased risk of overwhelming infections (Dahyot-Fizelier et al., 2013; Sinwar, 2014). Thus, proper immunization might be effective to prevent the risk of OPSI in splenectomized and asplenic patients. The low or retarded expression of *IgM* after vaccination in our study might provide theoretical support for a vaccination strategy in splenectomized and asplenic patients. IgZ is the only teleost Ig isotype with a specialized mucosal immune response function in zebrafish, and it shows a rapid induction after bacterial infection in gill and skin (Hu et al., 2010; Ji et al., 2021). In this study, *IgZ/IgZ2* were slightly increased at 10 d in gill and skin, and there was no significant difference in expression of *IgZ/IgZ2* in WT and congenitally asplenic zebrafish after vaccination (**Figure S7**). One possible explanation is that intraperitoneal injection of an inactivated vaccine might not able to reduce high expression of the *IgZ/IgZ2* gene. Further study will be conducted to study the effects of congenital asplenia on the mucosal immune response of skin and gill by pathogen challenge.

In summary, we present findings related to the molecular mechanism of systemic immune response in congenitally asplenic zebrafish and provide an in-depth understanding of spleen function in controlling immune homeostasis. To the best of our knowledge, we are the first to perform comparative transcriptome analysis to study the effects of congenital asplenia on innate and adaptive immune response by using a congenitally asplenic zebrafish model. Congenital asplenia might impair efferent neural connections from the brain to the spleen, inactivating the splenic anti-inflammatory reflex that, in turn, induces excessive cytokine/chemokine release and inflammatory damage and results in an impaired immune response.

## Data Availability Statement

The datasets presented in this study can be found in online repositories. The names of the repository/repositories and accession number(s) can be found below: https://www.ncbi.nlm.nih.gov/, PRJNA688352; https://www.ncbi.nlm.nih.gov/, PRJNA688655.

## Ethics Statement

The animal study was reviewed and approved by Institutional Animal Care and Use Committee (IACUC) of Southwest University.

## Author Contributions

Conceptualization, LX and YL. Methodology, LX and RW. Validation, LX and ZC. Resources, YL. Data curation, LX, ZC, and HG. Writing—original draft preparation, LX. Writing—review and editing, RW and YL. Visualization, LX. Supervision, YL. Funding acquisition, YL and RW. All authors have read and agreed to the published version of the manuscript. All authors contributed to the article and approved the submitted version.

## Funding

The research was supported by the Ecological Fishery Technological System of Chongqing Municipal Agricultural and Rural Committee under Grant (No. 4322000101) and Natural Science Foundation of Chongqing (cstc2020jcyj-msxmX0526).

## Conflict of Interest

The authors declare that the research was conducted in the absence of any commercial or financial relationships that could be construed as a potential conflict of interest.

## Supplementary Material

Supplementary Figure 1 PCA among samples.

Supplementary Figure 2 The number of DEGs for different groups. WT-0h, WT zebrafish without vaccination; Mut-0h, congenitally asplenic zebrafish without vaccination; WT-6h, WT zebrafish with vaccination sampled at 6 h; Mut-6h, congenitally asplenic zebrafish with vaccination sampled at 6 h; WT-10d, WT zebrafish with vaccination sampled at 10d; Mut-10d, congenitally asplenic zebrafish with vaccination sampled at 10 d.

Supplementary Figure 3 The DEGs involved in the TNF signaling pathway were annotated by KEGG in Mut-6h_vs_Mut-0h.

Supplementary Figure 4 The DEGs involved in the RIG-I-like receptor signaling pathway were annotated by KEGG in Mut-6h_vs_Mut-0h.

Supplementary Figure 5 The heat map with the expression pattern of inflammatory cytokines/chemokines at 0, 6 h and 10 d.

Supplementary Figure 6 The differentially expressed genes involved in the P53 signaling pathway were annotated by KEGG in Mut-6h_vs_Mut-0h.

Supplementary Figure 7 Expression profiles of *IgZ and IgZ2* after vaccination in gill and skin. WT animals and congenitally asplenic zebrafish sampled at 0 h were used as controls. The data were normalized to the expression level of WT-0h. The data are reported as the mean ± SEM. Different letters (a, b, c, etc.) indicate significant differences (*p* < .05).
