# Supplementary material for: Congenital Asplenia Interrupts Immune Homeostasis and Leads to Excessive Systemic Inflammation in Zebrafish
Source: Front Cell Infect Microbiol. 2021 Jun 28;11:668859. doi: 10.3389/fcimb.2021.668859 (PMC8274418; doi:10.3389/fcimb.2021.668859)
Supplement: Supplementary Figure 1 — PCA among samples. [file DataSheet_1.zip › Table S1.docx]

**Table S1**. Sequences of primers used in present study

| Primer name | Primer sequence (5’ to 3’) |
| --- | --- |
| Q*-Il1β-*F: | TGGACTTCGCAGCACAAAATG |
| Q*-Il1β-*R: | GTTCACTTCACGCTCTTGGATG |
| Q*-Il6-*F: | TGACCTCAGTCCTGGTGAAC |
| Q*-Il6-*R | TCGATCATCACGCTGGAGAA |
| Q*-tnfa-*F: | TACCGCTGGTGATAGTGTCC |
| Q*-tnfa-*R | CTGGGTCTTATGGAGCGTGA |
| Q*-tnfβ-*F: | CTGGCATGTGATGAAGCCAA |
| Q*-tnfβR*: | AGTGAATGGCAGCCTTTGTG |
| Q*-nfkb1-*F | AGGCCAAAGACACTGTTCGG |
| Q*-nfkb1-*R | GGAAAGGTTGTGGGGTCCAT |
| Q*-nfkb2-*F | AACAAGACGCAAGGAGCCCA |
| Q*-nfkb2-*R | CTGTCTCTTGCACAAAGGGC |
| Q*-ifnγ-*F | AATGACAGCGTGGATGAAGC |
| Q*-ifnγ-*R | GCTCAAACAAAGCCTTTCGC |
| Q*-il10-*F | TGGAGACCATTCTGCCAACA |
| Q*-il10-*R | GCATTTCACCATATCCCGCT |
| Q*-ch25h-*F | GTCCTTCTGGATGCCCTGTC |
| Q*-ch25h-*R | GGTTGTAGAGGGAGAGTGCG |
| Q*-cxcr3.3-*F | GGAGGAGCAAGGTCCACTTC |
| Q*-cxcr3.3-*R | AGAAACAAAGAGTCTCTCGGTCA |
| Q*- cxcl18b-*F | CTCTCGTGAATCGTGCTCGC |
| Q*- cxcl81b-*R | TGCAGTAATTGGCCCTGCTG |
| Q*- c3a.1-*F | CGATGCAGGGCTGATGTTTG |
| Q*- c3a.1-*R | TACTGACCAGCCAGTGTTGC |
| Q*- c3a.6-*F | CCCGAGTGTGTCATCCCTTC |
| Q*- c3a.6-*R | GTTCACACGTGTAGCCCAGT |
| Q*- c4-*F | TGCGGTCTCAGTTTTGGACA |
| Q*- c4-*R | ACCACCAGGGGTGAAATACG |
| Q*- ccl20a.3-*F | GAGTCGGATTTCAGCGTGTG |
| Q*- ccl20a.3-*R | TCTTGCCTCCGTTTGTGTGG |
| Q*- cxcl8a-*F | GAAAGCCGACGCATTGGAAA |
| Q*- cxcl8a-*R | TTAACCCATGGAGCAGAGGG |
| Q- *MHCII-*F: | TGACTCAACTGTCCGTGATA |
| Q- *MHCII-*R: | CCATTAGCCATCTCCATAGTG |
| Q- *IgM-*F: | GTGTTTGTGACTTGGCTTG |
| Q- *IgM -*R: | CACCCGTCCACTCTGAATT |
| Q- *IgZ -*F | CACCCAGCATTCTACAGCAAAC |
| Q- *IgZ -*R | GTCGGTACAAGAACCAAACTCAG |
| Q- *IgZ2 -*F | CAGAATGGAGCAAGCCTGAC |
| Q- *IgZ -*R | TAACTGTGCCCTCTTGGTGTATT |
| Q*-ef1α*-F | GCTTCTCTACCTACCCTCCTCT |
| Q*-ef1α*-R | CACCACCGATTTTCTTCTCA |
