# Supplementary material for: Congenital Asplenia Interrupts Immune Homeostasis and Leads to Excessive Systemic Inflammation in Zebrafish
Source: Front Cell Infect Microbiol. 2021 Jun 28;11:668859. doi: 10.3389/fcimb.2021.668859 (PMC8274418; doi:10.3389/fcimb.2021.668859)
Supplement: Supplementary Figure 1 — PCA among samples. [file DataSheet_1.zip › Table S2.docx]

**Table S2. The significantly regulated pathways induced by vaccination in zebrafish**

| **WT-6h_vs_WT-0h** | | | | | |
| --- | --- | --- | --- | --- | --- |
| id | Description | Significant | Annotated | Pvalue | Qvalue |
| ko03008 | Ribosome biogenesis in eukaryotes | 21/894 | 41/3957 | 5.32E-05 | 0.007194 |
| ko00480 | Glutathione metabolism | 18/894 | 33/3957 | 6.24E-05 | 0.007194 |
| ko00980 | Metabolism of xenobiotics by cytochrome P450 | 14/894 | 24/3957 | 0.00016 | 0.012215 |
| ko03050 | Proteasome | 21/894 | 45/3957 | 0.000291 | 0.012215 |
| ko04210 | Apoptosis | 38/894 | 100/3957 | 0.00031 | 0.012215 |
| ko04620 | Toll-like receptor signaling pathway | 26/894 | 61/3957 | 0.000353 | 0.012215 |
| ko04211 | Longevity regulating pathway - mammal | 32/894 | 81/3957 | 0.00041 | 0.012215 |
| ko00051 | Fructose and mannose metabolism | 13/894 | 23/3957 | 0.000424 | 0.012215 |
| ko04380 | Osteoclast differentiation | 33/894 | 85/3957 | 0.000486 | 0.012452 |
| ko04978 | Mineral absorption | 15/894 | 29/3957 | 0.000554 | 0.012765 |
| ko00680 | Methane metabolism | 13/894 | 24/3957 | 0.000736 | 0.015416 |
| ko04630 | Jak-STAT signaling pathway | 30/894 | 80/3957 | 0.00167 | 0.032079 |
| **Mut-6h_vs_Mut-0h** | | | | | |
| id | Description | Significant | Annotated | Pvalue | Qvalue |
| ko03008 | Ribosome biogenesis in eukaryotes | 21/870 | 41/3957 | 3.45E-05 | 0.006384 |
| ko04115 | p53 signaling pathway | 23/870 | 48/3957 | 5.78E-05 | 0.006384 |
| ko04210 | Apoptosis | 38/870 | 100/3957 | 0.000171 | 0.008831 |
| ko00480 | Glutathione metabolism | 17/870 | 33/3957 | 0.000178 | 0.008831 |
| ko04620 | Toll-like receptor signaling pathway | 26/870 | 61/3957 | 0.000222 | 0.008831 |
| ko04211 | Longevity regulating pathway - mammal | 32/870 | 81/3957 | 0.000243 | 0.008831 |
| ko04380 | Osteoclast differentiation | 33/870 | 85/3957 | 0.000286 | 0.008831 |
| ko04668 | TNF signaling pathway | 29/870 | 72/3957 | 0.00032 | 0.008831 |
| ko00980 | Metabolism of xenobiotics by cytochrome P450 | 13/870 | 24/3957 | 0.000556 | 0.013663 |
| ko04622 | RIG-I-like receptor signaling pathway | 17/870 | 36/3957 | 0.000666 | 0.014713 |
| ko04630 | Jak-STAT signaling pathway | 30/870 | 80/3957 | 0.001051 | 0.02009 |
| ko04068 | FoxO signaling pathway | 39/870 | 112/3957 | 0.001091 | 0.02009 |
| ko00051 | Fructose and mannose metabolism | 12/870 | 23/3957 | 0.001409 | 0.02396 |
| ko04922 | Glucagon signaling pathway | 28/870 | 77/3957 | 0.002604 | 0.041122 |
