# Supplementary material for: Congenital Asplenia Interrupts Immune Homeostasis and Leads to Excessive Systemic Inflammation in Zebrafish
Source: Front Cell Infect Microbiol. 2021 Jun 28;11:668859. doi: 10.3389/fcimb.2021.668859 (PMC8274418; doi:10.3389/fcimb.2021.668859)
Supplement: Supplementary Figure 1 — PCA among samples. [file DataSheet_1.zip › Table S5.docx]

Table S5. The significantly up-regulated GO terms involved in adaptive immunity in WT-10d_vs_WT-0h

| GO.ID | Term | Ontology | Significant | Annotated | Pvalue | Qvalue |
| --- | --- | --- | --- | --- | --- | --- |
| GO:0046649 | lymphocyte activation | biological process | 27/759 | 133/20731 | 3.20E-13 | 1.66E-10 |
| GO:0042110 | T cell activation | biological process | 17/759 | 66/20731 | 1.50E-10 | 5.74E-08 |
| GO:0002250 | adaptive immune response | biological process | 14/759 | 83/20731 | 1.80E-06 | 0.0002202 |
| GO:0051251 | positive regulation of lymphocyte activation | biological process | 12/759 | 62/20731 | 2.10E-06 | 0.0002534 |
| GO:0051249 | regulation of lymphocyte activation | biological process | 14/759 | 85/20731 | 2.40E-06 | 0.0002819 |
| GO:0072676 | lymphocyte migration | biological process | 9/759 | 43/20731 | 2.10E-05 | 0.0016369 |
| GO:0002455 | humoral immune response mediated by circulating immunoglobulin | biological process | 10/759 | 54/20731 | 2.30E-05 | 0.0017616 |
| GO:0019724 | B cell mediated immunity | biological process | 10/759 | 58/20731 | 4.30E-05 | 0.0029822 |
| GO:0016064 | immunoglobulin mediated immune response | biological process | 10/759 | 58/20731 | 4.30E-05 | 0.0029822 |
| GO:0002460 | adaptive immune response based on somatic recombination of immune receptors built from immunoglobuli... | biological process | 11/759 | 72/20731 | 5.80E-05 | 0.0037289 |
| GO:0048247 | lymphocyte chemotaxis | biological process | 8/759 | 38/20731 | 5.70E-05 | 0.0037289 |
| GO:0042113 | B cell activation | biological process | 10/759 | 61/20731 | 6.80E-05 | 0.0043089 |
| GO:0002449 | lymphocyte mediated immunity | biological process | 10/759 | 66/20731 | 0.00013 | 0.0075332 |
| GO:0050671 | positive regulation of lymphocyte proliferation | biological process | 5/759 | 15/20731 | 0.00014 | 0.0077555 |
| GO:0042102 | positive regulation of T cell proliferation | biological process | 5/759 | 15/20731 | 0.00014 | 0.0077555 |
| GO:0019814 | immunoglobulin complex | cellular component | 9/799 | 51/21244 | 0.00011 | 0.0089444 |
| GO:0031294 | lymphocyte costimulation | biological process | 4/759 | 9/20731 | 0.00019 | 0.0096735 |
| GO:0031295 | T cell costimulation | biological process | 4/759 | 9/20731 | 0.00019 | 0.0096735 |
| GO:0050870 | positive regulation of T cell activation | biological process | 5/759 | 16/20731 | 0.0002 | 0.0100091 |
| GO:0050853 | B cell receptor signaling pathway | biological process | 9/759 | 58/20731 | 0.00024 | 0.0116149 |
| GO:0002377 | immunoglobulin production | biological process | 6/759 | 27/20731 | 0.00036 | 0.0166888 |
| GO:0050864 | regulation of B cell activation | biological process | 8/759 | 50/20731 | 0.00043 | 0.0195229 |
| GO:0050868 | negative regulation of T cell activation | biological process | 5/759 | 20/20731 | 0.00064 | 0.0267162 |
| GO:0050672 | negative regulation of lymphocyte proliferation | biological process | 4/759 | 12/20731 | 0.0007 | 0.0282826 |
| GO:0042130 | negative regulation of T cell proliferation | biological process | 4/759 | 12/20731 | 0.0007 | 0.0282826 |
| GO:0050863 | regulation of T cell activation | biological process | 6/759 | 33/20731 | 0.00112 | 0.0419786 |
| GO:0030098 | lymphocyte differentiation | biological process | 7/759 | 46/20731 | 0.00132 | 0.0432334 |
| GO:0050871 | positive regulation of B cell activation | biological process | 7/759 | 46/20731 | 0.00132 | 0.0432334 |
| GO:0050670 | regulation of lymphocyte proliferation | biological process | 5/759 | 23/20731 | 0.00126 | 0.0432334 |
| GO:0042129 | regulation of T cell proliferation | biological process | 5/759 | 23/20731 | 0.00126 | 0.0432334 |
| GO:0042098 | T cell proliferation | biological process | 5/759 | 24/20731 | 0.00155 | 0.0485851 |
