# Supplementary figures and images for: Congenital Asplenia Interrupts Immune Homeostasis and Leads to Excessive Systemic Inflammation in Zebrafish
Source: Front Cell Infect Microbiol. 2021 Jun 28;11:668859. doi: 10.3389/fcimb.2021.668859 (PMC8274418; doi:10.3389/fcimb.2021.668859)

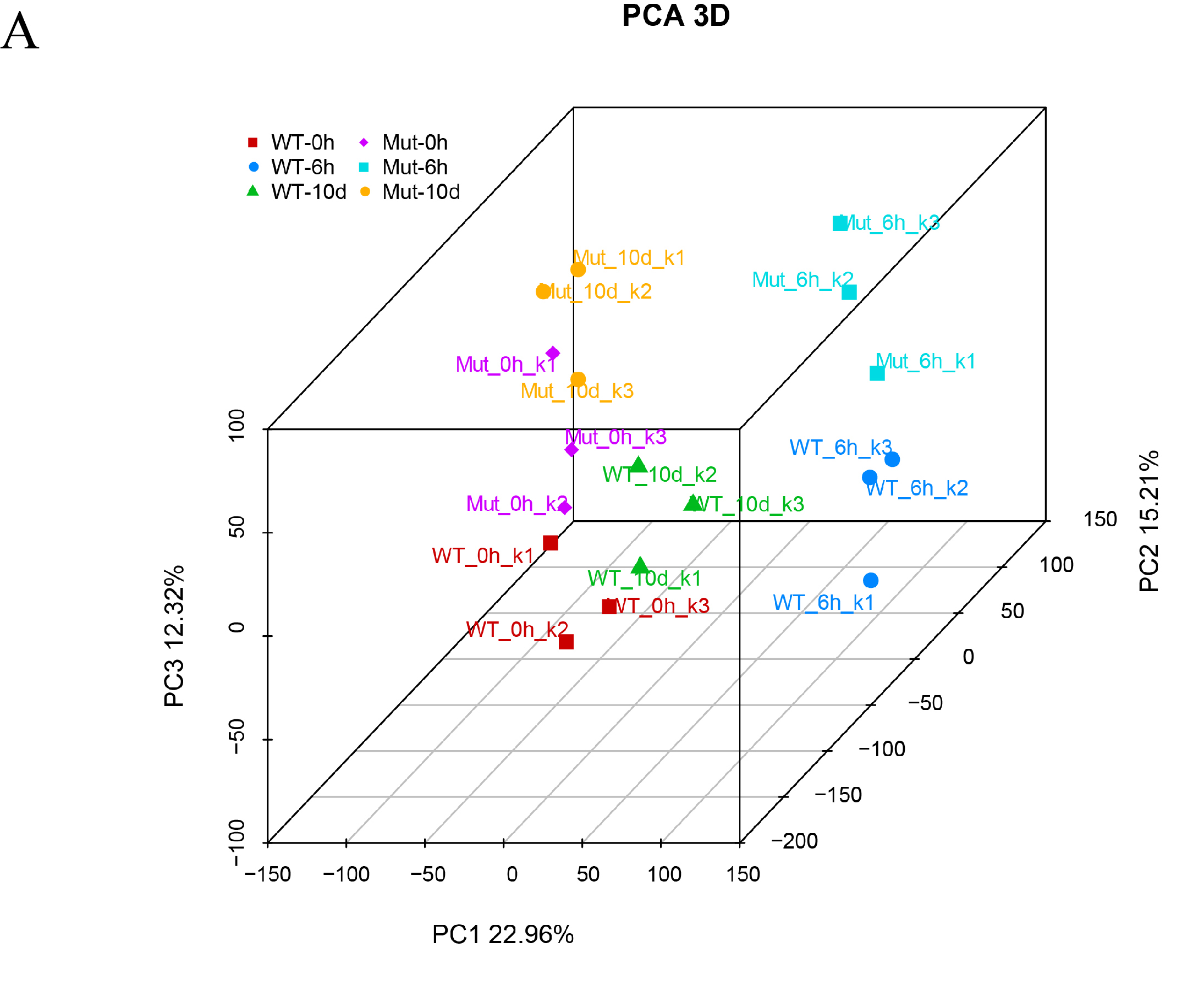

Supplement: Supplementary Figure 1 — PCA among samples. [file DataSheet_1.zip › Figure S1.jpg]

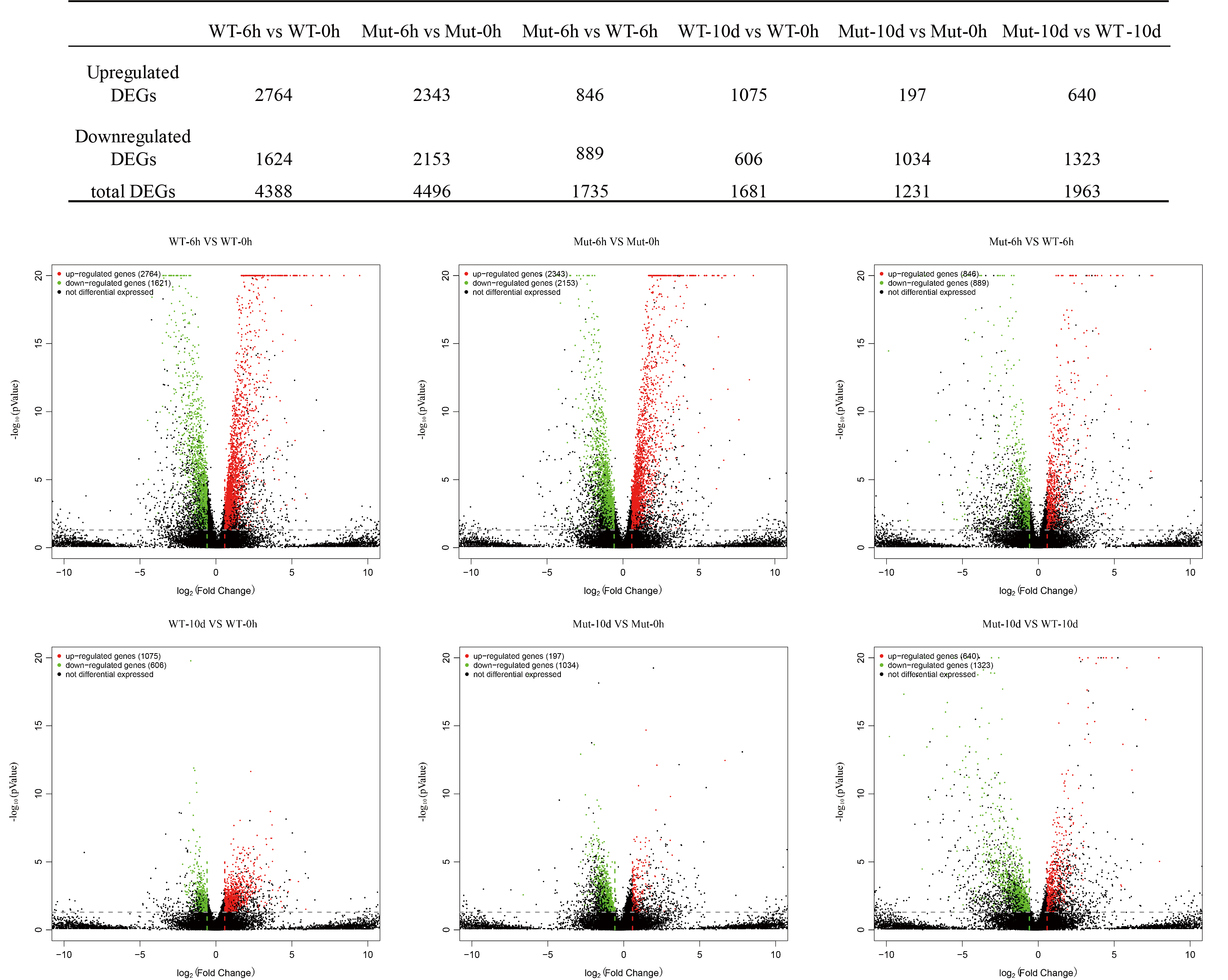

Supplement: Supplementary Figure 1 — PCA among samples. [file DataSheet_1.zip › Figure S2.jpg]

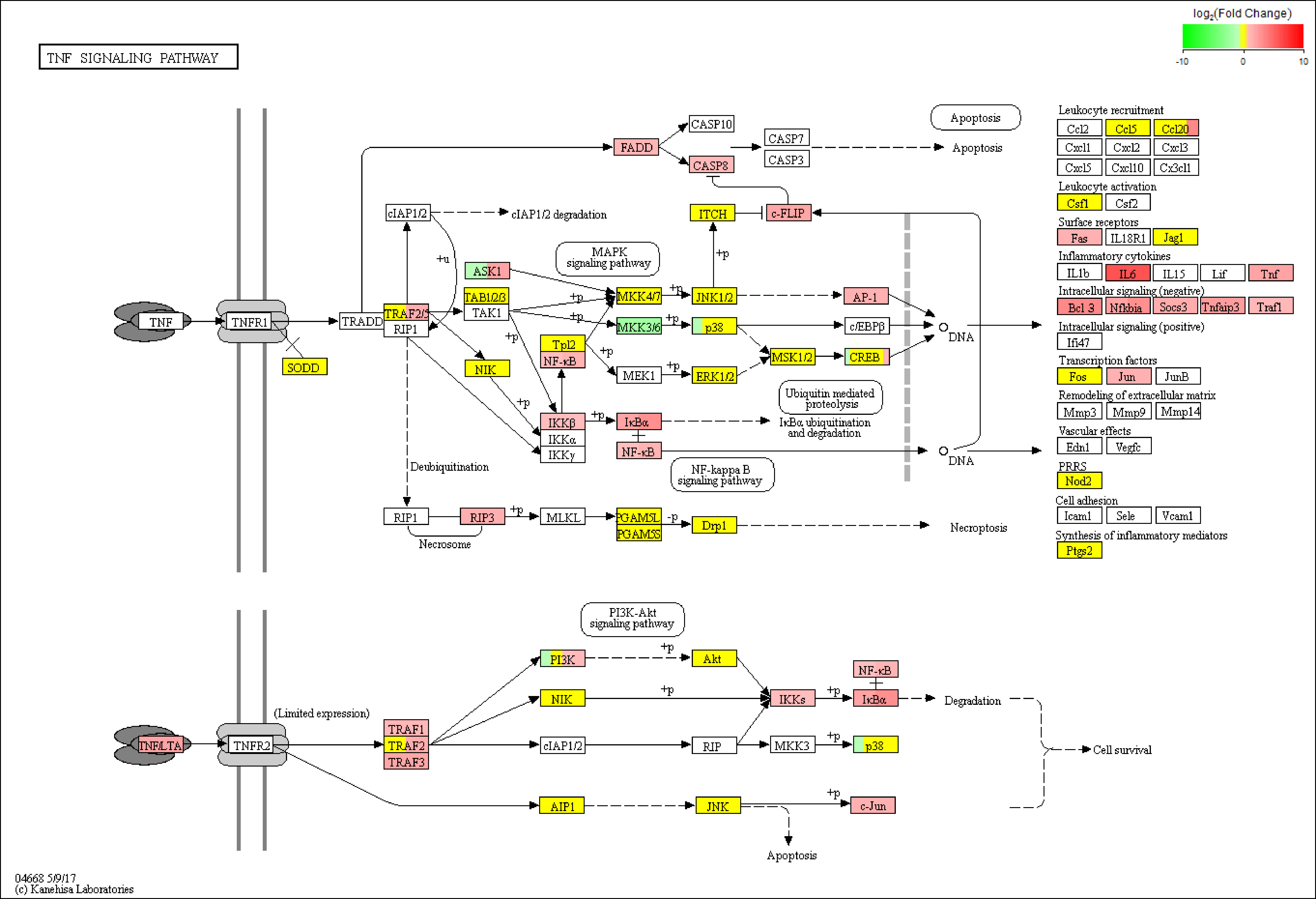

Supplement: Supplementary Figure 1 — PCA among samples. [file DataSheet_1.zip › Figure S3.jpg]

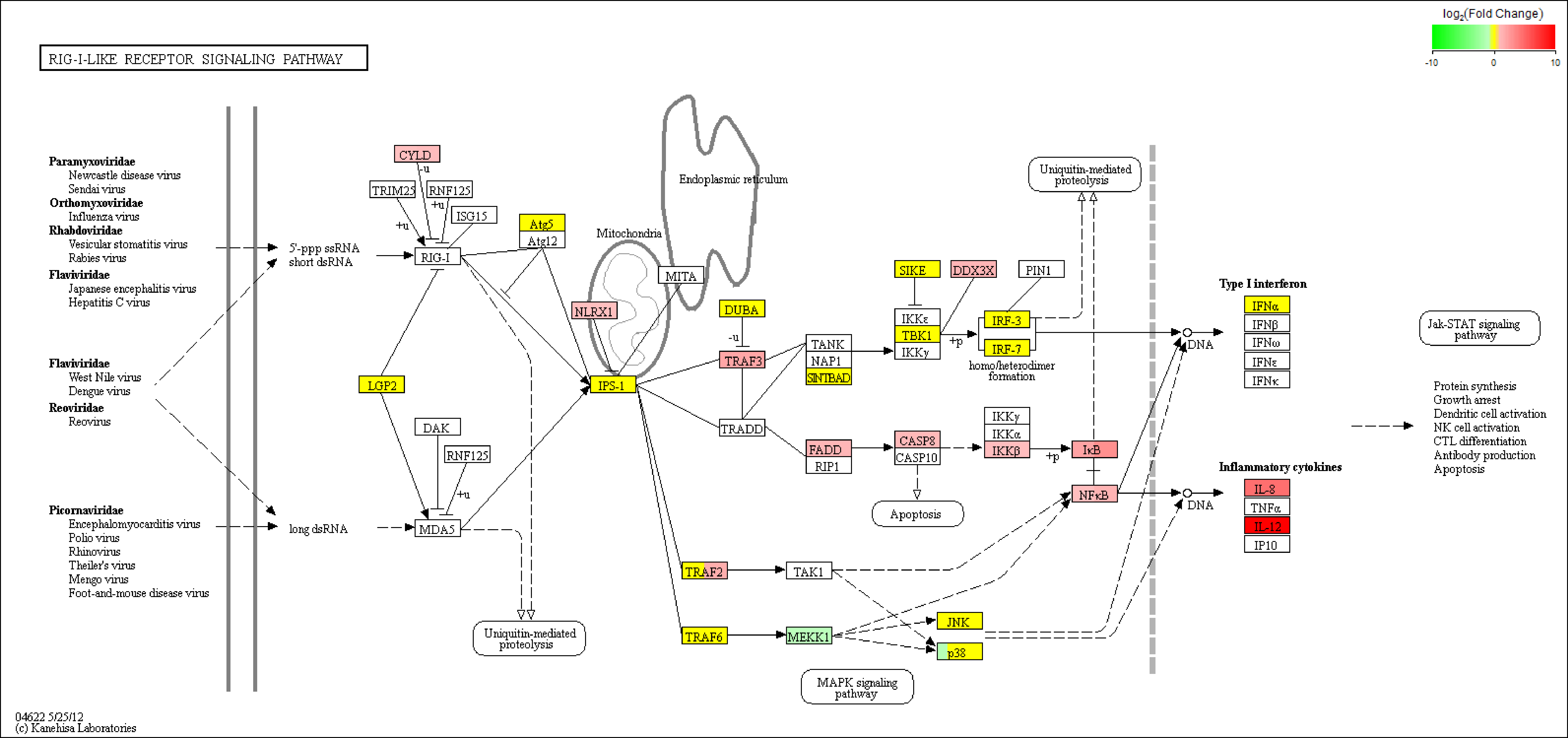

Supplement: Supplementary Figure 1 — PCA among samples. [file DataSheet_1.zip › Figure S4.jpg]

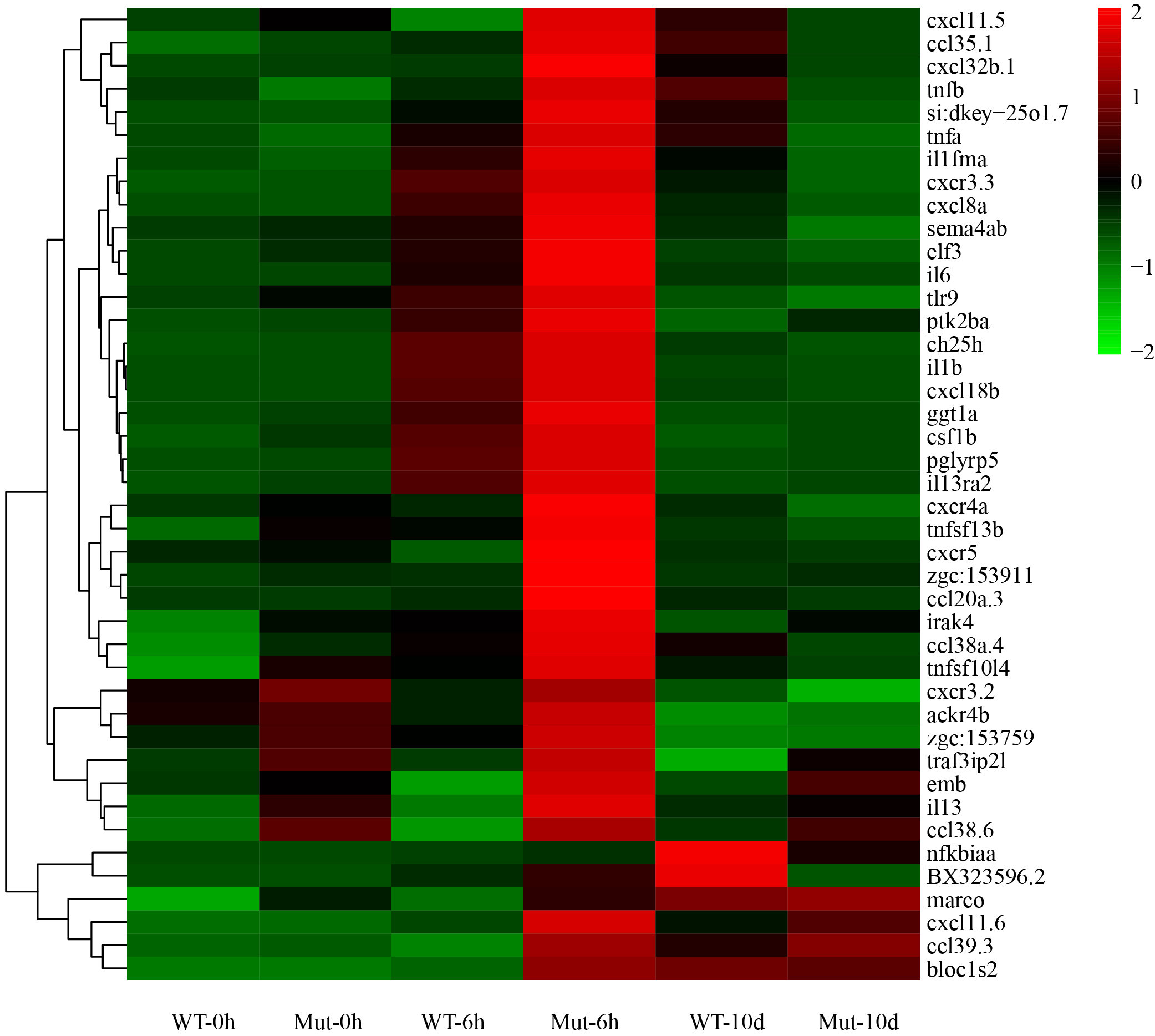

Supplement: Supplementary Figure 1 — PCA among samples. [file DataSheet_1.zip › Figure S5.jpg]

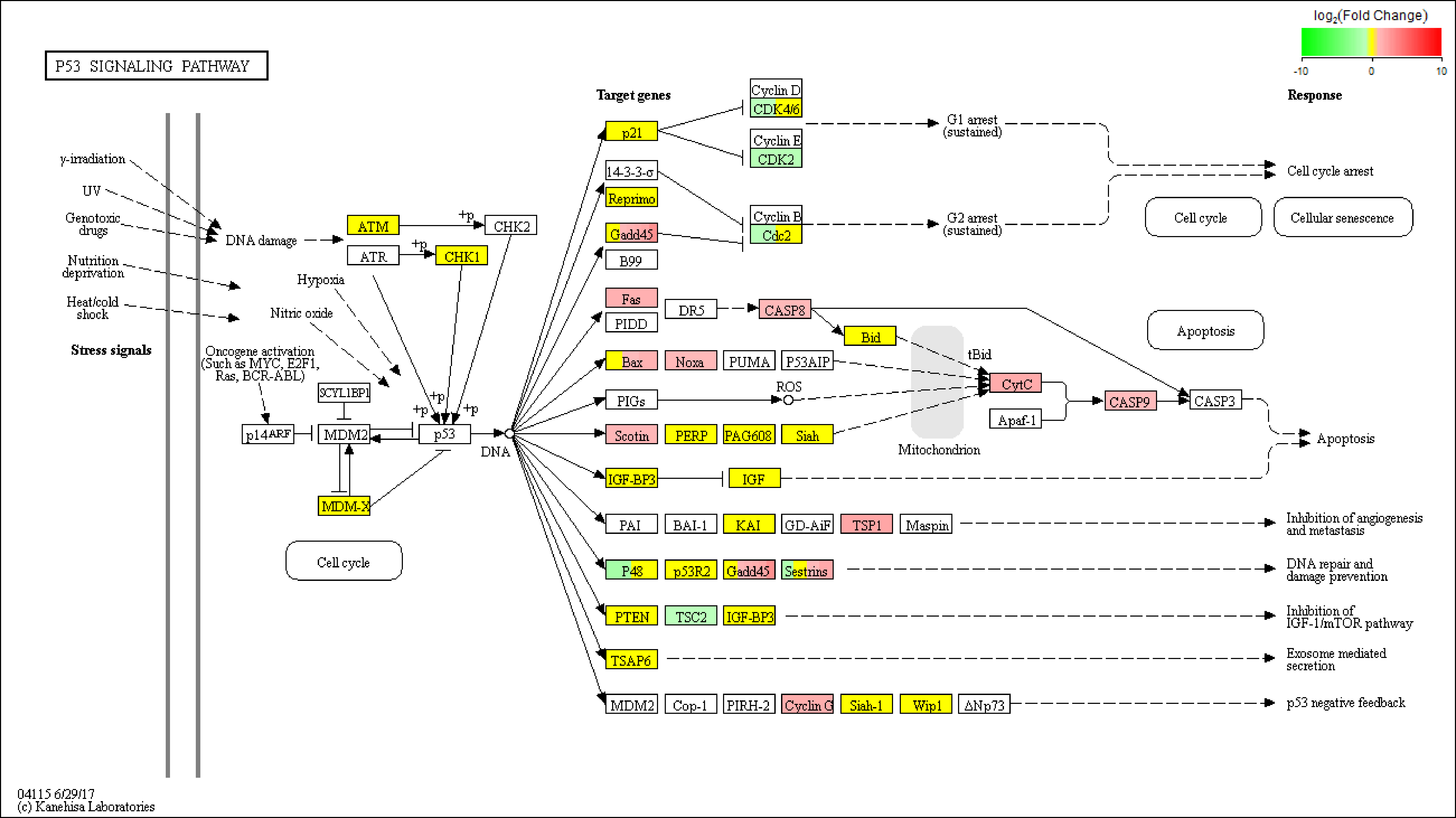

Supplement: Supplementary Figure 1 — PCA among samples. [file DataSheet_1.zip › Figure S6.jpg]

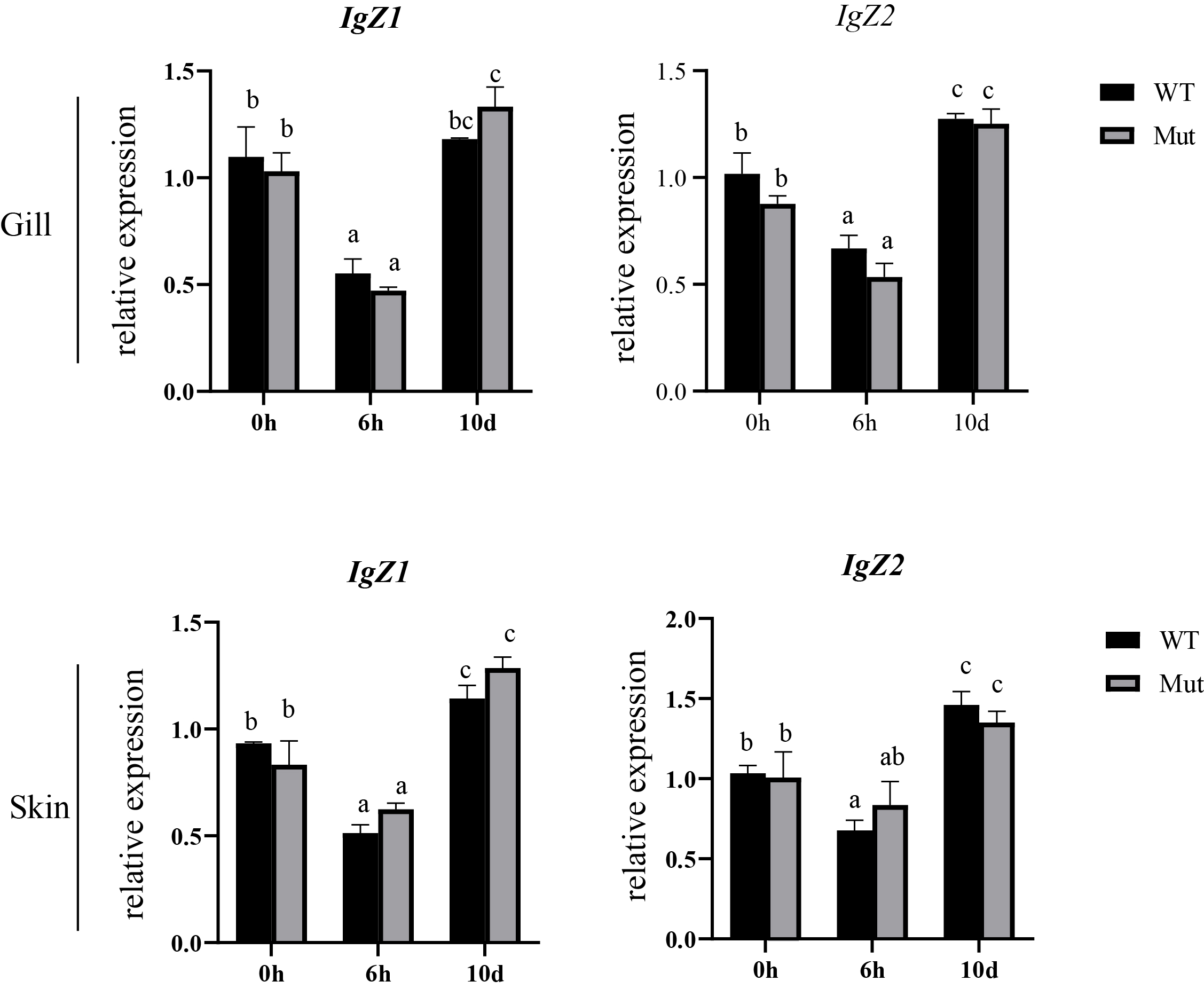

Supplement: Supplementary Figure 1 — PCA among samples. [file DataSheet_1.zip › Figure S7.jpg]
